# Nearest Neighbour Propensity Score Matching and Bootstrapping for Estimating Binary Patient Response in Oncology: A Monte Carlo Simulation

**DOI:** 10.1038/s41598-020-57799-w

**Published:** 2020-01-22

**Authors:** Tine Geldof, Dusan Popovic, Nancy Van Damme, Isabelle Huys, Walter Van Dyck

**Affiliations:** 1 KU Leuven, Department of Pharmaceutical and Pharmacological Sciences, Research Centre for Pharmaceutical Care and Pharmaco-economics O&N II, 3001 Leuven, Belgium; 2 Vlerick Business School, Healthcare Management Centre, Reep 1, 9000 Ghent, Belgium; 3 KU Leuven, Department of Electrical Engineering, Stadius Centre for Dynamical Systems, Signal Processing and Data Analytics, Kasteelpark Arenberg 10, 3001 Leuven, Belgium; 4 Belgian Cancer Registry, Koningsstraat 215, 1210 Brussels, Belgium

**Keywords:** Medical research, Epidemiology, Risk factors, Applied mathematics, Statistics

## Abstract

Nearest Neighbour (NN) propensity score (PS) matching methods are commonly used in pharmacoepidemiology to estimate treatment response using observational data. Unfortunately, there is limited evidence on the optimal approach for accurately estimating binary treatment response and, more so, to estimate its variance. Bootstrapping, although commonly used to accurately estimate variance, is rarely used together with PS matching. In this Monte Carlo simulation-based study, we examined the performance of bootstrapping used in conjunction with PS matching, as opposed to different NN matching techniques, on a simulated dataset exhibiting varying levels of real world complexity. Thus, an experimental design was set up that independently varied the proportion of patients treated, the proportion of outcomes censored and the amount of PS matches used. Simulation results were externally validated on a real observational dataset obtained from the Belgian Cancer Registry. We found all investigated PS methods to be stable and concordant, with k-NN matching to be optimally dealing with the censoring problem, typically present in chronic cancer-related datasets, whilst being the least computationally expensive. In contrast, bootstrapping used in conjunction with PS matching, being the most computationally expensive, only showed superior results in small patient populations with long-term largely unobserved treatment effects.

## Introduction

Estimating treatment effects in real-world clinical practice becomes increasingly important in domains like oncology, due to the high complexity of cancers and to recent developments of targeted medicines and immune-oncology drugs^1^. However, cancer registries, which are often used in epidemiological studies while monitoring changes in disease prevalence and investigating differences in incidence rates, only collect data at the time of diagnosis and, as such, do not contain any longitudinal information^2^. Therefore, estimating patient responses to a treatment, which could be based on tumour growth and/or toxic events, becomes very difficult. In those cases, patient-level response to a treatment can be based on overall survival (OS) as identified in clinical trials. Patient-level treatment effect can then be derived using the Propensity Score (PS) modelling technique proposed by Rosenbaum *et al*. (1983) for estimating average treatment effects^3,4^, a common method used in pharmacoepidemiology. In this technique, the PS is the likelihood of a patient being assigned to a treatment (treatment status Z = 1 for treated vs. Z = 0 for control patients), conditional on observed covariates ***X*** prior to the application of the treatment. It forms the basis for matching treated and control patients who have a similar PS value, that is, are nearest neighbours^3,4^. Henceforward, changes in patients’ individual Survival Gain (SG) (i.e. the difference between OS for treated and matched control patient), for each treated patient forms an indication for the response of the patient to the treatment.

Yet, caution should be taken when using Nearest Neighbour (NN) PS matching. First, one assumes that the OS of both treated and matched control patient(s) are exact and hence that the time of death is observed. In reality, patients get lost to follow up or are still alive during data collection, censoring the actual survival time and hence causing a problem when estimating the SG. This is especially true for chronic cancers like colorectal cancer featuring a median OS extending to a couple of years with treatment^5–8^. Secondly, there is still some controversy in the literature as to how variance and standard error of treatment effects should be calculated^9–11^. Providing a prediction error seems to be challenging as the variance will be high for one-by-k NN PS matching, on the one hand due to the small sample sizes when k is very low (approaching one) and on the other hand due to high patient heterogeneity when k is very high, that is, approaching the entire patient population.

Bootstrap-based methods, relying on random resampling, have been proposed in a few simulation studies and found to be effective for estimating variance of average patient treatment effects (ATE)^9–13^. Although rarely used in conjunction with PS matching, the technique is well-known for its ability to accurately measure variances of estimations in analytical difficult cases such as small datasets^14^, and shows therefore promise for use in uncertainty surrounding (individual) treatment effects.

The main goal of this research is to assert which technique is the best for reducing the censoring problem and estimating the variance of the estimated SG in the context of predicting binary patient-level treatment response. Simulation-based approaches are well suited for this purpose, because these simulate true values which can then be compared to estimates generated from different PS matching approaches under varying conditions. Therefore, we examine the performance of three different state-of-the-art techniques on simulated datasets with different levels of heterogeneity. In particular, we compare k-NN, weighted k-NN and complex bootstrapping as described by Austin (2014) in series of Monte Carlo simulations. Finally, we further validate our findings on four case studies of metastatic colorectal patients treated with targeted medicines.

Counterintuitively, we found complex bootstrapping not to outperform k-NN or weighted k-NN methods when estimating survival gain variance in highly heterogeneous patient populations. However, from the aflibercept case featuring a small amount of patients assigned to the treatment with highly censored survival times, we did observe the bootstrap method to have favourable estimations. As expected, although computationally being the most expensive, bootstrapping outperformed other methods estimating variance in fairly small datasets.

## Background: Propensity Score Matching and Boostrapping

This section presents the PS matching technique for estimating treatment effect and describes how different greedy NN algorithms^14^ and the bootstrapping method^9–13^ can be used to mitigate the censoring problem and to estimate uncertainty on individual treatment effects. Each of the matching algorithms uses matching with replacement, so that each control unit can be matched to multiple treated units.

### PS NN matching and treatment effect

In NN PS matching, each treated patient is matched to one or more patients from the control group based on the closest ${\rm{PS}}=Pr({Z}_{i}=1|{X}_{i})$ value^3,4,15^. In principle, any regression technique can be used to develop the propensity model as long as it provides reasonable fit to the data. It is not necessary that the chosen technique produces calibrated probabilities as units are matched on a score^16^. Optimal selection of variables *X*_*i*_ is based on observed variables which affect the outcome of interest, because this is associated with better PS estimations^17^.

Let *T* and *C* be the set of *N*^*T*^ treated and *N*^*C*^ control patients respectively and $O{S}_{i}^{T}$ and $O{S}_{i}^{C}$ the observed continuous outcomes of the treated and control units, respectively. Denote by *C*(*i*) the set of ${N}_{i}^{C}$ control units matched (using NN based on PS scores) to the treated unit *i* ∈ *T*. Define the weights ${w}_{ij}=\frac{1}{{N}_{i}^{C}}$ if *j* ∈ *C*(*i*) and ${w}_{ij}=0$ otherwise. We define the subject-specific treatment effect (STE) for the treated, derived from the ATE^13,18^, as the estimation of the subject-specific survival gain $S{G}_{i}^{s}$:

1 $$\widehat{S{G}_{i}^{s}}=O{S}_{i}^{T}-\sum _{j\in C(i)}{w}_{ij}O{S}_{j}^{C}$$

With variance

$$\widehat{{V}_{i}^{s}}=var(\widehat{S{G}_{i}^{s}})=var(O{S}_{i}^{T})+\sum _{j\in C(i)}{{w}_{ij}}^{2}var(O{S}_{j}^{C})$$

Following oncology guidelines and depending on disease severity, patients can be labelled with ‘response’ whenever their SG is longer than a threshold of *λ* months.

### One-by-one matching

The most common implementation of PS matching in practice is one-by-one matching, in which pairs of treated and control units are formed. Using one-by-one nearest neighbour PS matching $({N}_{i}^{C}=1)$, one treated unit *i* ∈ *T* is matched to one control unit *j* ∈ *C*. When the OS of treated, control or both are censored, the estimated SG^s^ will be highly uncertain (see Supplementary Material). Hence, for those matched pairs where censoring is problematic, the binary response-label based on the estimated SG^s^ becomes highly uncertain. For those cases, the SG cannot be assessed if any of the following conditions apply:

- when *j* ∈ *C*(*i*) and *i* ∈ *T* are censored
- when *j* ∈ *C*(*i*) is censored and $\widehat{S{G}_{i}^{s}}\ge \,\lambda$
- when *i* ∈ *T* censored and $\widehat{S{G}_{i}^{s}} < \,\lambda$

Denote ${\kappa }_{ij}=NA$ when *i* and *j* are censored or when *j* is censored and $O{S}_{i}^{T}-O{S}_{j}^{C}\ge \lambda$ and ${\kappa }_{ij}=1$ otherwise. Define *ρ*_*i*_ = 1 when *i* is observed or when *i* is censored but $O{S}_{i}^{T}-{\kappa }_{ij}O{S}_{j}^{C}\ge \lambda$ and *ρ*_*i*_ = *NA* otherwise. Formula (1) becomes:

2 $$\widehat{S{G}_{i}^{s}}={\rho }_{i}O{S}_{i}^{T}-{\kappa }_{ij}O{S}_{j}^{C}$$

3 $$\widehat{{V}_{i}^{s}}=var(\widehat{S{G}_{i}^{s}})=0$$

with $O{S}_{i}^{T}\,$assumed to be a constant. We can make a crude estimation of the $S{G}_{i}^{s}$ variance by taking the variance of the entire control set *C*, i.e. ${V}_{i}^{s}=var(O{S}^{C})$.

### One-by-k matching

Using one-by-k nearest neighbour PS matching (${N}_{i}^{C}=k$ = 50), one treated unit *i* ∈ *T* is matched to k nearest control units. Labelling for matched units subject to the censoring problem cannot be estimated if any of the following conditions are satisfied:

- when $\forall j\in C(i):j$ and *i* ∈ *T* are censored
- when $\forall j\in C(i):j$ is censored and $\widehat{S{G}_{i}^{s}}\ge \lambda$
- when $\,i\in T$ censored and $\widehat{S{G}_{i}^{s}} < \lambda$

When none of these conditions are met the response label of treated unit *i* ∈ *T* can be estimated. However, if $\exists j\in C(i):j$ is censored, *j* cannot contribute to the estimation of this label.

Define ${\delta }_{j,C(i)}=1$ when *j* is observed or when $\forall j\in C(i):j$ is censored and ${\delta }_{j,C(i)}=0$ otherwise, ${\kappa }_{C(i)}=NA$ when $\forall j\in C(i):i$ and *j* are censored, ${\kappa }_{C(i)}=1$ when $\exists j\in C(i):j$ is observed or when $\forall j\in C(i):j$ is censored but $O{S}_{i}^{T}-\sum _{j\in C(i)}{w}_{ij}O{S}_{j}^{C} < \lambda$. Denote ${\rho }_{i}=1$ when *i* is observed or when *i* is censored but $O{S}_{i}^{T}-\sum _{j\in C(i)}{\kappa }_{C(i)}{\delta }_{j}{w}_{ij}O{S}_{j}^{C}\ge \lambda$, given that the summation can be calculated under the conditions given by the definition of ${\kappa }_{C(i)}$, and ${\rho }_{i}=NA$ otherwise.

4 $$\widehat{S{G}_{i}^{s}}={\rho }_{i}O{S}_{i}^{T}-\sum _{j\in C(i)}{\kappa }_{C(i)}{\delta }_{j,C(i)}{w}_{ij}O{S}_{j}^{C}$$

5 $$\widehat{{V}_{i}^{s}}=\sum _{j\in C(i)}{{\kappa }_{C(i)}}^{2}{{\delta }_{j,C(i)}}^{2}{{w}_{ij}}^{2}var{(O{S}_{j}^{C})}_{C(i)}$$

### One-by-k weighted matching

In formula (2–5) all the *k* nearest neighbour units *j* ∈ *C* included in the calculation (i.e. for which censoring is not a problem) have weights ${w}_{ij}=\frac{1}{{N}_{i}^{C}}$ and all others weight zero, meaning that all matched control units have equal contribution to the calculated mean. This can be generalized to weighted NN PS matching, where the contribution of *j* ∈ *C*(*i*) to the mean depends on how similar the PSs are of subjects *i* and *j*, i.e. on the distance ${d}_{ij}=P{S}_{i}-P{S}_{j}$ (with minimal and maximal values equal to 0 and 1 respectively). Using an exponential distance function, the previous defined weights can be generalized to ${w}_{ij}=\frac{exp(-\alpha {d}_{ij})}{\sum _{j\in C(i)}exp(-\alpha {d}_{ij})}$ if *j* ∈ *C*(*i*) and *w*_*ij*_ = 0 otherwise^19^. The value of *α* is set to 5 to ensure weights close to zero for maximal distances while having large enough differences in weights for small distances. Matched control units with equal PS as the treated unit contribute to the mean with a weight equal to one, while matched control units with distance approaching one contribute only with a weight approaching zero.

### Matching through bootstrapping

Using the complex bootstrap method as described by Austin (2014), *b* bootstrap samples are drawn from the original control group with sample size equal to the control group^9^. In each of the bootstrap samples, the PS model is estimated, and one-by-one PS matching is performed for creating matched pairs, forming the set *C*(*i*) of control units (${N}_{i}^{C}$ = *b*) matched to the treated unit *i* ∈ *T*. In this k-NN bootstrapping method, one treated patient can be matched multiple times to the same control patient, i.e. *j* ∈ *C*(*i*) can occur multiple times in *C*(*i*), lowering the heterogeneity of the matched sets. The estimation of the subject-specific gain in survival $\widehat{S{G}_{i}^{s}}$ and its variance $\widehat{{V}_{i}^{s}}$ can be calculated as given by Eqs. (4) and (5) in one-by-k matching^14^.

## Material and Methods

We used simulated datasets with three levels of patient heterogeneity to examine the performance of the different matching techniques over a series of Monte Carlo simulations. There, performance was evaluated based on their ability to estimate the individual SG under these three scenarios. In this section, we describe the design of the datasets and the Monte Carlo simulations. The results were externally validated by examination of case studies for treated metastatic colorectal patients.

### Simulated data generation

Data was simulated in R following the data-generating process described by Austin (2014), generating 1000 patients with 10 baseline covariates *X*_1_ − *X*_10_, of which seven affecting treatment selection (*X*_1_ − *X*_7_) and OS outcome (*X*_3_ − *X*_10_)^9^. Very weak, weak, moderate, strong and very strong effects of the covariates on treatment selection and OS outcome is introduced by the regression coefficients ${\alpha }_{VW}=log\,log\,(1,25),\,{\alpha }_{W}=log\,log(1,5),{\alpha }_{M}=log\,log(2),{\alpha }_{S}=log\,log(4)$ and ${\alpha }_{VS}=log(8)$. PSs ${p}_{i}=Pr({Z}_{i}=1|{X}_{i})$ were determined using logistic regression, following:

$$\begin{array}{ccc}logit{({p}_{i})}_{L} & = & {\alpha }_{0,treat,L}+{\alpha }_{VW}{x}_{1}+{\alpha }_{W}{x}_{2}+{\alpha }_{VW}{x}_{3}+{\alpha }_{W}{x}_{4}+{\alpha }_{VW}{x}_{5}+{\alpha }_{W}{x}_{6}+{\alpha }_{M}{x}_{7},\\ logit{({p}_{i})}_{M} & = & {\alpha }_{0,treat,M}+{\alpha }_{W}{x}_{1}+{\alpha }_{M}{x}_{2}+{\alpha }_{S}{x}_{3}+{\alpha }_{W}{x}_{4}+{\alpha }_{M}{x}_{5}+{\alpha }_{S}{x}_{6}+{\alpha }_{VS}{x}_{7},\\ logit{({p}_{i})}_{H} & = & {\alpha }_{0,treat,H}+{\alpha }_{M}{x}_{1}+{\alpha }_{S}{x}_{2}+{\alpha }_{M}{x}_{3}+{\alpha }_{S}{x}_{4}+{\alpha }_{M}{x}_{5}+{\alpha }_{S}{x}_{6}+{\alpha }_{VS}{x}_{7}\end{array}$$

for low, medium and high heterogeneity respectively. Treatment status was generated from a Bernoulli distribution on the subject-specific PS ${p}_{i}:{Z}_{i} \sim Be({p}_{i})$, through which the intercept *α*_0,*treat*_ indirectly affects the proportion of patients treated in the simulation. The OS outcome was generated as described by Bender (2005) and Austin (2014)^9,12^, that is, based on the linear predictor

$$\begin{array}{ccc}L{P}_{L} & = & {\beta }_{treat,L}Z+{\alpha }_{W}{x}_{4}+{\alpha }_{VW}{x}_{5}+{\alpha }_{W}{x}_{6}+{\alpha }_{M}{x}_{7}+{\alpha }_{VW}{x}_{8}+{\alpha }_{W}{x}_{9}+{\alpha }_{VW}{x}_{10},\\ L{P}_{M} & = & {\beta }_{treat,M}Z+{\alpha }_{W}{x}_{4}+{\alpha }_{M}{x}_{5}+{\alpha }_{S}{x}_{6}+{\alpha }_{VS}{x}_{7}+{\alpha }_{W}{x}_{8}+{\alpha }_{M}{x}_{9}+{\alpha }_{S}{x}_{10},\\ L{P}_{H} & = & {\beta }_{treat,H}Z+{\alpha }_{S}{x}_{4}+{\alpha }_{M}{x}_{5}+{\alpha }_{S}{x}_{6}+{\alpha }_{VS}{x}_{7}+{\alpha }_{M}{x}_{8}+{\alpha }_{S}{x}_{9}+{\alpha }_{M}{x}_{10}\end{array}$$

for low, medium and high heterogeneity respectively, using the formula $OS=(\frac{-log(u)}{\lambda {e}^{LP}}){}^{\frac{1}{2}}$, with *u* a random number from the uniform distribution and 𝜆 equal to 0.00002. The conditional hazard ratio *exp*(*β*_*treat*_) was fixed to 0.8. The true SG for the treated *SG*_*i*_ was generated from the OS outcome as produced by the linear predictors for *Z*_*i*_ = 1 and 0: $S{G}_{i}|({Z}_{i}=1)=O{S}_{i}|({Z}_{i}=1)-O{S}_{i}|({Z}_{i}=0)$. From this, the corresponding average true SG, i.e. the “*true ATE*”$\,\overline{SG}$, and the variance of the true SG, i.e. the “*true variance*” *V*, is calculated. The censoring status of the subjects’ survival was drawn from a Binominal distribution given the probability of being censored ${p}^{\ast }:{c}_{i} \sim Binom({p}^{\ast })$.

### Case study data

Patients were collected from the Belgian Cancer Registry (BCR), a population based cancer registry. We used ICD-10 codes (C18 up to and including C20) to select 10426 metastatic colorectal patients (stadium IV carcinoma) diagnosed between 2006 and 2014 with vital status information updated until July 1, 2017 (Table 1). Patients were classified in five groups according to their targeted treatment assignment: 2784, 845, 308 and 31 patients received bevacizumab, cetuximab, panitumumab, and aflibercept respectively. 6458 patients were not treated with the targeted medicine and were classified as the control group (irrespective of radiotherapeutic and/or chemotherapeutic treatments). Of these five groups, 26% (731), 15% (127), 11% (35), 52% (16) and 15% (965) had censored survival, respectively.

**Table 1:** Outcomes for each treatment resulting from the different NN PS matching techniques (k = 5).

Product	PS NN techn.	ATE ($\overline{{\boldsymbol{S}}{{\boldsymbol{G}}}^{{\boldsymbol{s}}}}$, months)	$\hat{{\boldsymbol{V}}}$	$\overline{{{\boldsymbol{V}}}^{{\boldsymbol{s}}}}$	${\boldsymbol{var}}({{\boldsymbol{V}}}^{{\boldsymbol{s}}})$	C
Bevacizumab *(2784 patients)*	1:5	8.00	567.51	310.21	2.45e + 5	4.6%
	weighted 1:5	7.97	566.04	312.30	2.51e + 5	4.6%
	5 *bootstrap*	*7.39*	*600.79*	*282.97*	*2.3*5*e* + 5	*5.2%*
Cetuximab *(845 patients)*	*1:5*	*7.38*	*477.96*	*261.86*	*1.54e* + *5*	*1.8%*
	weighted 1:5	7.31	482.54	264.88	1.56e + 5	1.8%
	5 bootstrap	6.33	534.84	293.23	2.18e + 5	2.5%
Panitumumab *(308 patients)*	*1:5*	*11.25*	*359.27*	*282.25*	*2.48e* + *5*	*1.6%*
	weighted 1:5	10.96	329.42	309.92	2.60e + 5	1.6%
	5 bootstrap	9.71	453.27	276.24	2.75e + 5	1.6%
Aflibercept *(31 patients)*	1:5	2.28	323.71	424.96	3.16e + 5	23%
	weighted 1:5	2.56	320.35	428.04	3.32e + 5	23%
	*5 bootstrap*	*3.18*	*340.35*	*337.58*	*2.50e* + *5*	*19%*

OS, the RCT’s primary endpoint, was used as the main indication of treatment effect. Selected variables were taken from the full standard set of variables nationally collected by the BCR and Inter Mutualistic Agency, which were further limited for relevance by BCR oncologists.

The data set consisted of (a) the patient’s OS and censoring status; (b) (historical) treatment paths i.e. radiotherapeutic and/or chemotherapeutic treatment and treatment with the four targeted medicines; and (c) patient and tumour characteristics, i.e. age, sex, tumour differentiation grade, topography, tumour location (left/right), total amount of tumours, WHO performance score at diagnosis and TNM classification. Multiple imputation was used for handling missing data for PS-relevant variables assuming data was missing completely at random^20–23^.

### Analysis on simulated data

Using the data-generating process described above, three types of heterogeneity were simulated by using the regression coefficients denoting very weak to very strong impact on treatment selection and survival, which were iterated 1000 times. For each of these heterogeneity types (low, medium and high), three factors were varied: the proportion of patients treated (given no censoring), the proportion of outcomes censored (given 20% of patient treated) and the number of nearest neighbours used in matching (given 20% of patient treated and 20% of outcomes censored). (See Supplementary Materials for more information). For all these scenarios and datasets, the PS is estimated using a logistic regression model^3,4^, with selected observed variables being those affecting the survival time (*X*_3_ − *X*_10_)^16^.The three PS NN matching techniques (k-NN, weighted k-NN and complex bootstrapping described above) are performed to estimate the STE, i.e. the estimated subject-specific gain in survival $\widehat{S{G}_{i}^{s}}$, and STE variance $\widehat{{V}_{i}^{s}}$ for each treated unit *i* ∈ *T*, given by formula (4 and 5). These are then investigated for the different PS NN methods by calculating their means over all units, $\,\overline{S{G}^{s}}\,$the mean STE) and $\overline{{V}^{s}}$ (the mean STE variance), and comparing the latter with the simulated “*true variance*” *V*. We propose the PS NN method with the smallest relative difference $\frac{{\delta }_{var}}{V}=(V-\overline{{V}^{s}})$)/V the best estimator of the true variance. Lastly, the variance of $\widehat{{V}^{s}}$, i.e. *var*(*V*^*s*^), is compared between the PS NN methods, as well as the proportion of patients subject to the label-censoring problem as defined by the rules of formula (4)-(5). These analyses were carried out across the 1000 iterations of the Monte Carlo simulation conducted in R. Therefore, results of these analysis are reported as averaged values over the iterations.

## Simulation Results

The following section describes the results of the label-censoring problem and the variance estimations for the three PS NN methods on the simulated datasets with low, medium and high heterogeneity.

### Impact of number of units matched

**Figure 1:**
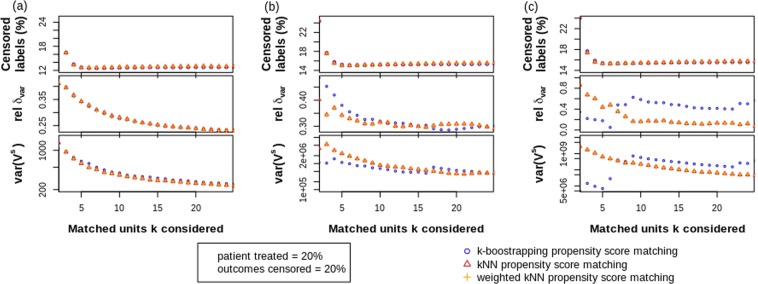
Monte Carlo simulation results in function of the number of NN matched (given 20% of patient treated and 20% of outcomes censored) for (**a**) low heterogeneity; **(b**) medium heterogeneity and (**c**) high heterogeneity.

The relative difference *δ*_*var*_ between the true variance *V* and the mean estimated STE variance $\overline{{V}^{s}}$ together with the resulting variance of the STE variance *var*(*V*^*s*^) and the proportion of labels censored are reported in Fig. 1 for varying amount of NN units *k* considered during matching. The three panels show the different levels of heterogeneity.

As expected, the amount of predicted labels that are censored decrease with increasing amount of matched units *k* considered during the three PS NN matching methods for all heterogeneity sets, this at a similar pace until *k* = 5. Hence all methods perform equally well for solving the label-censoring problem, regardless of heterogeneity.

Similarly, no difference is found between the methods for estimating variance in low heterogeneous groups unless for computational complexity using bootstrapping. However, for increasing heterogeneity, we observe the bootstrap method to have less accurate predictions of variances, showing higher relative differences *δ*_*var*_ although lower variances of $\widehat{{V}^{s}}$ for small *k*. Hence, for high heterogeneity the bootstrap method would be inferior to the k-NN matching methods based on both accuracy and computational complexity, while for low heterogeneity the bootstrap method is inferior on computational complexity alone.

### Impact of proportion of outcomes censored

**Figure 2:**
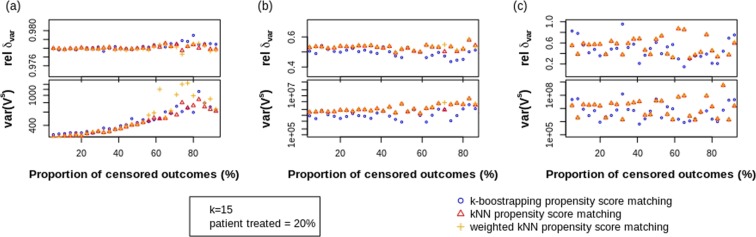
Simulation results in function of the proportion of OS outcomes censored (given k = 15 NN used in matching and 20% of patients treated) for (**a**) low heterogeneity; (**b**) medium heterogeneity and (**c**) high heterogeneity.

The relative difference *δ*_*var*_ between the true variance *V* and the mean estimated STE variance $\overline{{V}^{s}}$ and the resulting variance of the STE variance *var*(*V*^*s*^) are reported in Fig. 2 for varying amount of outcomes OS censored. The three panels show the different levels of heterogeneity.

We see the relative difference *δ*_*var*_ to be quite unaffected by the proportion of OS outcomes censored for all heterogeneity sets. Hence, the estimation of $\overline{{V}^{s}}$ remains constant, even though increased outcomes censored means less units *j* ∈ *C*(*i*) contribute to the estimation of ${V}_{i}^{s}$ for every unit *i* ∈ *T*. However, we can verify that this has an effect on the accuracy of this estimation, because the variances of $\widehat{{V}^{s}}$ have an increasing trend for low heterogeneity. This trend disappears for higher heterogeneity because of both *δ*_*var*_ and especially *var*(*V*^*s*^) fluctuate. For all levels of heterogeneity, the bootstrap method would be inferior to the k-NN matching method based on computational complexity.

### Impact of proportion of patients treated

**Figure 3:**
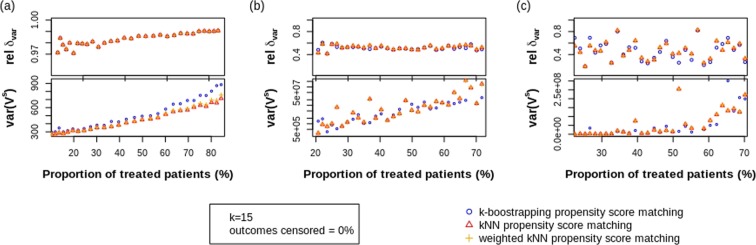
Simulation results in function of the proportion of patients treated (given k = 15 NN used in matching and 0% of outcomes censored) for (**a**) low heterogeneity; (**b**) medium heterogeneity and (**c**) high heterogeneity. The error bars have been omitted for clarity.

The relative difference *δ*_*var*_ between the true variance *V* and the mean estimated STE variance $\overline{{V}^{s}}$ and the resulting variance of the STE variance *var*(*V*^*s*^) are reported in Fig. 3 for varying amounts of patients treated in the population. The three panels show the different levels of heterogeneity.

As expected, *δ*_*var*_ increases and becomes more uncertain ($\widehat{{V}^{s}}$ variance increases) for increasing proportion of treated patients in all heterogeneity sets, as the control group *C* (the pool to which treated units are matched) becomes smaller. No difference is found between the different methods, except for a slightly higher $\widehat{{V}^{s}}$ variance for low heterogeneity. However, as for each proportion of treated units a different linear predictor was simulated, affecting the OS outcome of the dataset, we see *δ*_*var*_ and $\widehat{{V}^{s}}$ variance fluctuates, especially for high heterogeneity. Therefore, results for *δ*_*var*_ for the different PS NN methods are inconclusive.

## Case Study

**Figure 4:**
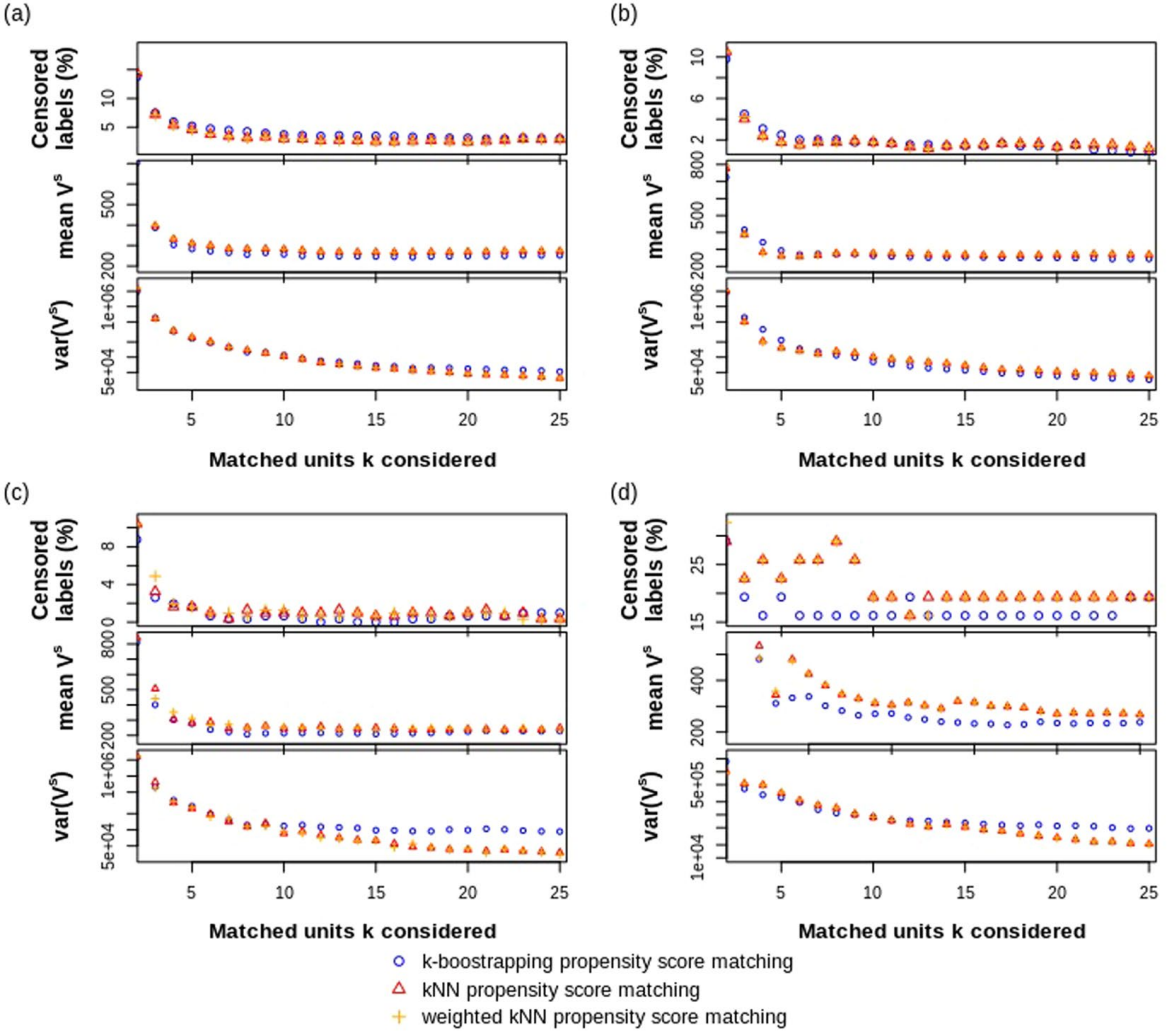
Case study comparison of (weighted) k-NN and bootstrap matching for (**a**) bevacizumab (2784 treated patients), (**b**) cetuximab (845 treated patients), (**c**) panitumumab (308 treated patients), and (**d**) aflibercept (31 treated patients). Shown are the number of SG outcomes being censored (top), the mean subject-specific treatment-effect (STE) variance (middle) and the variance of the STE variance (bottom).

In this section, the performance of the three different PS NN techniques are examined on a case study of metastatic colorectal cancer patients treated with bevacizumab, cetuximab, panitumumab or aflibercept as a targeted medicine. Numerical results of the one-by-one, one-by-25 (weighted) and 25-bootstrap PS NN matching techniques are depicted in Fig. 4 and Table 1.

The results show that the three methods are stable and concordant. Only for the aflibercept case, with a small treated population (31) and high amount of survival censoring (52% or 16 out of 31), we observe a difference between the k-NN techniques and the bootstrap method. Specifically, the censoring problem reduces dramatically with increasing *k* with lower estimated $\overline{{V}^{s}}$ and $\widehat{{V}^{s}}$ variance for the bootstrap method as opposed to the k-NN techniques.

## Discussion

Bootstrapping, a method commonly used to accurately estimate variance, is rarely used together with PS matching. In this Monte Carlo simulation-based study, we examined the performance of the complex bootstrap method, as described by Austin (2014), to estimate binary treatment response and variance in the domain of oncology. Specifically, the subject-specific survival gain (that is, the individual treatment effect) and its variance together with its ability to mitigate the problem of label-censoring, obtained from the individual treatment effect as a binary treatment response label, were the main factors under investigation. The Monte Carlo study was based on simulated datasets containing 1000 patients with varying levels of heterogeneity found in real world patient populations. Counterintuitively, we found that the estimation of survival gain variance in patient populations with a high patient heterogeneity does not benefit from using the complex bootstrapping method instead of (weighted) k-NN. Indeed, we expected the relevant matches to be small for increasing *k* and increasing heterogeneity, implying that k-NN PS matching would contain a large set of irrelevant matches for large *k*, as opposed to the complex bootstrapping method, which always matches a treated patient to the one closest control patient. As a consequence, while also taking into account the computational complexity we found the bootstrap method to not show to favourable results even for high heterogeneous patient populations. Additionally, no major differences were found between the k-NN and weighted k-NN method, because the resulting weights were approximately equal to one in most cases of the simulated data. While it can be argued that this behaviour would change if one chooses a value of the parameter of the exponential distance function that is better suited to the data at hand, we note that this parameter cannot be tuned in practice because, as opposed to that in simulation study, the real variance is unknown before estimation.

Applying these methods to four colorectal cancer treatments with varying amount of patients treated and unobserved outcomes, we found all three PS methods to be stable and concordant. From the analysis, we can conclude that the computationally cheapest method, being k-NN PS matching, should be used in most of the cases. However, for the aflibercept case, where a small amount of patients are assigned to the treatment while the majority of survival times are censored, we did observe the bootstrap method to have favourable estimations. This result was expected because bootstrapping is a statistical method often used for estimating variance in fairly small datasets^13^.

Note that some concerns may arise when using bootstrapping in conjunction with PS matching in observational studies. First, one specified PS model was used for each resampled control group in the analysis of this study, which may be inappropriate in high heterogeneous patient populations. However, identifying the best fitted model for each bootstrapped sample would be highly unpractical. Second, for comparison reasons, a low number of bootstrap samples was used equal to the number of matched control units in k-NN. Although this amount should provide a decent estimate^13^, a higher amount would be recommended in observational studies^13,23,24^.

Overall, given these findings, we suggest that the complex bootstrap method, while being computationally more expensive, should not be used for estimating subject-specific survival gain in large cohorts of treated and non-treated patients. However, this most computationally expensive method might show to be necessary when considering small patient populations with long-term and largely unobserved treatment effects.

## Supplementary information

Supplementary Materials.

## Data Availability

The case study data that support the findings of this study are available from the Belgian Cancer Registry but restrictions apply to the availability of these data, which were used under license for the current study, and so are not publicly available. Data are however available from the authors upon reasonable request and with permission of the Belgian Cancer Registry.
