# Supplementary material for: Nearest Neighbour Propensity Score Matching and Bootstrapping for Estimating Binary Patient Response in Oncology: A Monte Carlo Simulation
Source: Sci Rep. 2020 Jan 22;10:964. doi: 10.1038/s41598-020-57799-w (PMC6976708; doi:10.1038/s41598-020-57799-w)
Supplement: Supplementary file 1 — Supplementary Materials. [file 41598_2020_57799_MOESM1_ESM.docx]

**Nearest Neighbour Propensity Score Matching and Bootstrapping for Estimating Binary Patient** **Response in Oncology: A Monte Carlo Simulation – Supplementary Materials**

Tine Geldof, MSc1,2, Dusan Popovic, PhD3, Nancy Van Damme, PhD4, Isabelle Huys, PhD1*, Walter Van Dyck*, MA, MSc, PhD,2

1 KU Leuven, Department of Pharmaceutical and Pharmacological Sciences, Research Centre for Pharmaceutical Care and Pharmaco-economics O&N II, 3001 Leuven, Belgium.

2 Vlerick Business School, Healthcare Management Centre, Reep 1, 9000 Ghent, Belgium

3 KU Leuven, Department of Electrical Engineering, Stadius Centre for Dynamical Systems, Signal Processing and Data Analytics, Kasteelpark Arenberg 10, 3001 Leuven, Belgium.

4 Belgian Cancer Registry, Koningsstraat 215, 1210 Brussels, Belgium

1. **The label-censoring problem**

| **MATCHED PAIRS** $I \in T, J\in C\left( I \right)$ | ${OS}_{j}^{C}$ **available** | ${OS}_{j}^{C}$**censored** |
| --- | --- | --- |
| ${OS}_{i}^{T}$ **available** |  | *if SG^s^ ≥* $\lambda$ |
|  |  | *Else* |
| ${OS}_{i}^{T}$ **censored** | *if SG^s^ ≥* $\lambda$ |  |
|  | *Else* |  |

***Table 1. Representation of the label-censoring problem. White areas indicate no censoring problem, orange areas indicate problematic conditions.*** $\lambda$ ***is the SG threshold indicating treatment response when*** ${SG}_{i}^{T}\geq\lambda$***.***

## **R statistical software code for generating the simulated datasets: example of low heterogeneity**

# Random number seed set for each heterogeneity level, outside the Monte Carlo simulation.

set.seed(123)

N <- 1000 # Size of simulated patient population in each Monte Carlo iteration.

# Initiate values for each of the 10 baseline covariates $X_{1}-X_{10}$.

x.1 <- rnorm(N,0,1)

x.2 <- rnorm(N,0,1)

x.3 <- rnorm(N,0,1)

x.4 <- rnorm(N,0,1)

x.5 <- rnorm(N,0,1)

x.6 <- rnorm(N,0,1)

x.7 <- rnorm(N,0,1)

x.8 <- rnorm(N,0,1)

x.9 <- rnorm(N,0,1)

x.10 <- rnorm(N,0,1)

# Initiate values for very weak, weak, moderate, strong and very strong effects through regression coefficients.

beta.v.weak <- log(1.25)

beta.weak <- log(1.5)

beta.med <- log(2)

beta.strong <- log(4)

beta.v.strong <- log(8)

# Set the intercept in the treatment-selection model (treatment prevalence).

# The appropriate value of this intercept is found through iteration.

beta.0.treat <- scan("beta.0.treat.out")

# Generate treatment status for each subject.

# Note: different logit functions apply to generate the treatment status, depending on the level of heterogeneity.

# Note: when varying the proportion of patients treated (figure 3), this step is performed inside the for-loop.

logit.low..treat <- beta.0.treat + beta.v.weak*x.1 +beta.weak*x.2 + beta.v.weak*x.3 + beta.weak*x.4 +

beta.v.wea*x.5 + beta.weakh*x.6 + beta.med*x.7

p.treat <- exp(logit.low..treat)/(1 + exp(logit.low..treat))

treat <- rbinom(N,1,p.treat)

# Generate a survival outcome (time-to-event) for each subject. True hazard ratio is 0.8.

# Note: different linear predictor functions apply, depending on the level of heterogeneity.

beta.hr*treat <- log(0.8)

linpred <- beta.hr*treat + beta.weak*x.4 + beta.v.weak*x.5 + beta.weak*x.6 + beta.med*x.7 +

beta.v.weak*x.8 + beta.weak*x.9 + beta.v.weak*x.10

lambda <- 0.00002

nu <- 2

ranu <- runif(N,min=0,max=1)

surv.time <- ( -(log(ranu))/(lambda*exp(linpred)) )ˆ(1/nu)

# Set the amount of outcome censoring.

# Note: when varying the proportion of outcomes censored (figure 2), this step is performed inside the for-loop.

surv.status <- rep(1,0.8)

1. **Analysis on simulated data**


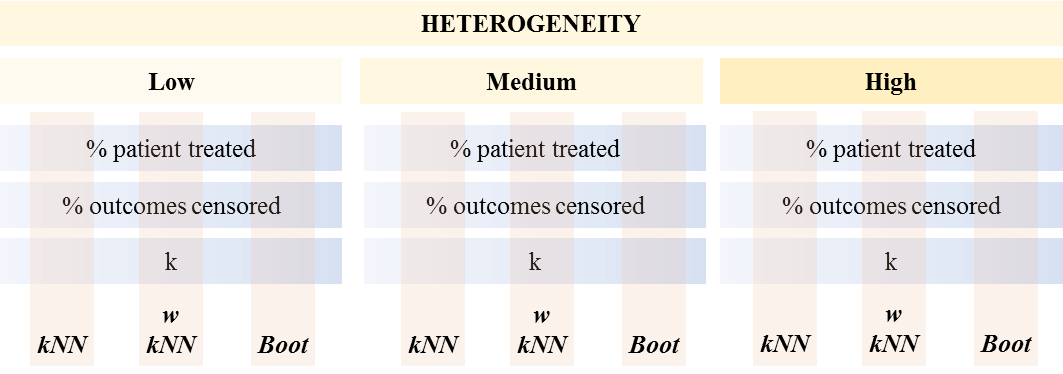


***Figure 1. Analysis overview on the simulated datasets. Three levels of patient heterogeneity are investigated. In each case, the three NN PS techniques are compared through a one-way sensitivity analysis, that is, by independently varying either the proportion of patients treated, the proportion of outcomes censored or number of nearest neighbours k with the other two of characteristics fixed.***


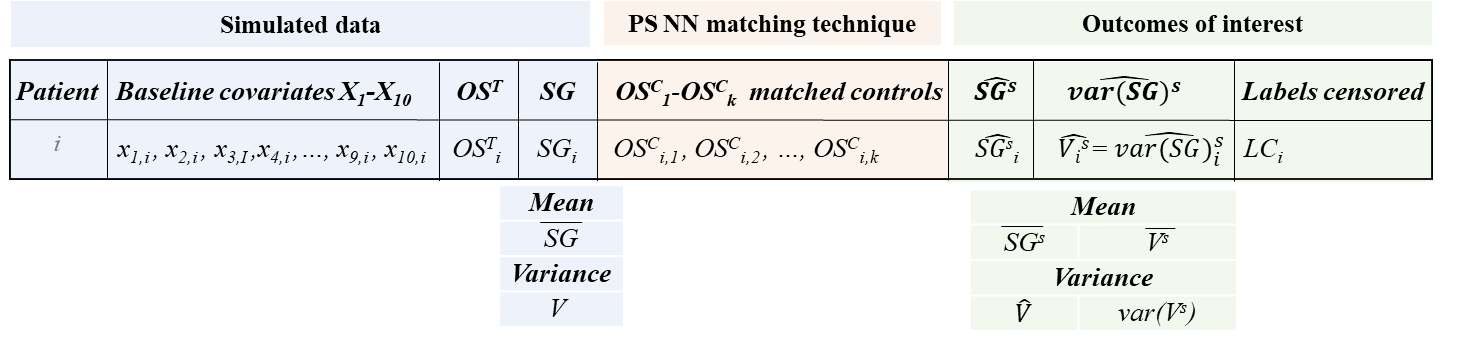

***Figure 2. General overview of the dataset in one iteration containing simulated baseline covariates X_1_-X_10_ and outcomes*** ${OS}^{T}$***and*** $SG$***, calculated matched covariates*** ${{OS}^{C}}_{1}$***-***${{OS}^{C}}_{k}$ ***and estimated outcomes of interest using PS NN matching:*** $\hat{{SG}^{s}}$***,*** $\hat{V^{s}}$ ***and label censoring.***
